# Use of domperidone in canine visceral leishmaniasis: gaps in veterinary knowledge and epidemiological implications

**DOI:** 10.1590/0074-02760180301

**Published:** 2018-10-18

**Authors:** Bruno L Travi, Guadalupe Miró

**Affiliations:** 1 University of Texas Medical Branch, Department of Internal Medicine - Division of Infectious Diseases, Galveston, Texas, USA; 2 Universidad Complutense de Madrid, Veterinary Faculty, Animal Health Department, Madrid, Spain

**Keywords:** domperidone, dogs, visceral leishmaniasis

## Abstract

A pivotal strategy to decrease the risk of visceral leishmaniasis in humans is to control the infection and disease progression in dogs, the domestic reservoir of *Leishmania infantum* (*L. chagasi*). Immunotherapy is a viable approach to treat sick dogs because cell-mediated immunity is the principal defense mechanism against *L. infantum*. Domperidone is an immune-stimulatory drug increasingly used in veterinary medicine as a prophylactic or immunotherapeutic agent. Domperidone treatment has shown to prevent overt disease or improve the clinical condition of infected dogs. However, veterinarians should be aware of the potential cardiotoxicity of domperidone when given together with drugs that inhibit CYP450s liver enzymes or those that prolong the QT interval. On the other hand, learning whether domperidone treatment significantly decreases dog infectivity to sand fly vectors is of capital importance since this result should have a palpable impact on the infection risk of humans living in regions endemic for visceral leishmaniasis.

A pivotal strategy to decrease the risk of visceral leishmaniasis in humans is to control the infection and disease progression in dogs, which are the principal reservoirs of *Leishmania infantum* (*L. chagasi*). Current efforts to control the dispersion of this important zoonosis focusing on dogs as complement of insecticide spraying have been unsuccessful leading, principally, to the expansion of canine visceral leishmaniasis (CVL) in South American countries. Instead of placing emphasis on the controversial strategy of culling infected dogs, new approaches to control transmission that are more humane need to be selected. Deltamethrin-impregnated collars and different spot-on insecticides have shown high repellent efficacy against sand fly vectors.^1^ In Europe, both preventive approaches are feasible while in Latin America they face logistical and economical hurdles for its implementation.^2^ ^,^ ^3^ Vaccines are showing promising results but additional field trials are required to understand better the epidemiological implications of its utilization, mostly in areas of high *L. infantum* transmission.^1^ ^,^ ^4^

Therefore, antileishmanial treatment of dogs is still a valid option since it delays disease progression that is associated with increased transmission to sand flies.^5^ ^,^ ^6^ ^,^ ^7^ The treatment of CVL is complex, frequently resulting in recurrent disease related with high parasite burdens.^8^ Combination of antimonials with allopurinol is currently the first line therapy used in most countries.^9^ ^,^ ^10^ In Brazil, miltefosine was approved for treatment of dogs infected with *L. infantum* in spite published information does not strongly support its utilization.^11^ ^,^ ^12^

Cell-mediated immunity is the principal defense mechanism to prevent or maintain *L. infantum* infection under control.^13^ ^,^ ^14^ For this reason, immunotherapy is being considered as a viable approach to treat sick dogs.^15^ Domperidone (Motilium®, Leishguard® or the generic drug) is an immune-stimulatory agent recommended in veterinary medicine as a prophylactic or immunotherapeutic agent, either as monotherapy or in combination with antileishmanial drugs.^16^ Although, few published studies are available detailing domperidone beneficial or untoward effects, its utilization is becoming increasingly popular in veterinary practice. A better understanding of its mode of action and drug interactions will maximize its efficacy and decrease the potential health risks to dogs at risk of *L. infantum* infection.

Domperidone is a benzimidazole derivative with selective dopamine D2 receptor antagonist activity.^17^ In humans, it is used as an anti-emetic, a gastro-kinetic drug or to increase milk production.^18^ In Veterinary medicine, domperidone could be used as a gastric pro-kinetic drug.^19^ The drug induces an increase of prolactin serum level as a secondary effect. This hormone, which is excreted from the pituitary gland and generated by lymphocytes, is considered to be a pro-inflammatory cytokine. It stimulates the cellular immunity (Th1) by increasing the production of INF-γ, IL2, IL12, and TNF-α.^20^

A clinical trial in 70 naturally infected dogs indicated that domperidone significantly improved the clinical condition of animals. Clinical improvement was observed in 86% (24/28) sick dogs, which was accompanied by stable antibody levels in 57.1 % of animals.^21^ Furthermore, at 90 days post-treatment, all infected dogs regardless of their initial clinical condition showed no clinical signs of disease and had negative or significantly lower antileishmanial antibody titers. The cell-mediated immunity improved in dogs that were at different disease stages as determined by cell proliferation assays and *Leishmania* skin testing.^21^ Unfortunately, no control group of untreated dogs was included in the study. This weakened the accuracy of domperidone results since it is known that a variable proportion of untreated dogs could remain asymptomatic or revert from sick to subclinical infection.^22^

Another clinical trial in an area highly endemic for CVL evaluated the capacity of domperidone to prevent development of disease.^23^ In this study, one group of healthy, seronegative dogs (n = 44) was treated with domperidone (0.5 mg/kg/day for 30 days, every 4 months) while a second similar group (n = 46) was left untreated. The clinical-serological evaluation done after exposure to two sand fly seasons showed that a significantly (p < 0.001) larger proportion of dogs treated with domperidone remained clinically healthy (89%) compared with untreated controls (52%). Authors defined this status as “protection” but the parasitological status of “protected dogs” was not assessed either by microscopy or molecular methods. Therefore, the impact of domperidone treatment on parasite burden in target organs (spleen, bone marrow, lymph nodes and/or skin) was not determined.

The label of veterinary domperidone (Leisguard™) does not recommend its use with cabergoline (dopamine agonist), dopamine and cimetidine, omeprazol or similar drugs. However, no trials to evaluate its interactions with other drugs have been performed. Consequently, we should not consider this drug as a supplement and have to be cautious with its administration. Personal experience (GM) have found some side effects associated with domperidone administration such as polyuria, dysorexia, vomiting and diarrhea. There are lingering questions regarding other aspects of domperidone therapy that require an in-depth discussion within the scientific and veterinary community. The treatment schedule for dogs recommended by the manufacturer of the veterinary product is 0.5 mg/kg/day for 30 days every three months, which is a low-dose regimen with few side effects. Since the recommendation of the manufacturer for dogs living in endemic areas is the administration of domperidone for three months each year, it would be important to gather more data on the potential short- and long-term clinical impact of this treatment in dogs of different age, breed and clinical condition.

The literature in humans indicated that some precautions need to be taken when using domperidone. A human population-based case-control study confirmed the higher risk of sudden cardiac death [OR (adj)] 11.4 (95% CI 1.99, 65.2) when taking high doses of domperidone, e.g. > 30 mg/day (> 0.4-0.5 mg/kg/day).^24^ While the recommended dose of domperidone in dogs falls within the safe range, it is important to consider two different situations that may lead to life-threatening arrhythmias. Domperidone is known to prolong the QT interval and this untoward effect could be synergized if other drugs with a similar side effect are co-administered to the animal.^25^ Table includes a partial list of drugs of veterinary use with known or suspected QT prolongation activity.

Another circumstance in which domperidone concentration could inadvertently increase is when dogs concomitantly receive drugs that have strong cytochrome P450 inhibitory activity. Domperidone has low bioavailability when given through the oral route but its absorption increases in the presence of CYP3A1 and P-gp inhibitors.^26^ Domperidone is principally metabolized by CYP3A4 (CYP3A12 or CYP3A26 are the canine orthologs) and the interaction with drugs that inhibit this detoxification enzyme may increase domperidone concentration exposing the animal to cardiotoxic levels (QT prolongation).^27^ Ketoconazole, itraconazole and erythromycin are examples of veterinary drugs that inhibit CYP450’s, and therefore should be avoided when dogs are under domperidone treatment.^28^ ^,^ ^29^ Other drugs with similar CYP450 inhibitory capacity are cimetidine, fluoroquinolones, chloramphenicol and phenobarbital.^28^ Before domperidone implementation, additional attention should be paid to conditions such as the age, breed and hormonal status of the animal. These variables have been associated with distinct detoxifying capacity of liver enzymes.^30^ Furthermore, the cardiological status, principally in older dogs or breeds prone to suffer heart conditions (i.e. boxer) must be considered.^31^

From the epidemiological standpoint, the impact of domperidone treatment on dog infectivity to sand fly vectors is still an open question. It is presumed that infected but clinically healthy dogs are at lower risk of transmitting *L. infantum* to sand flies compared with sick dogs. This has been demonstrated in studies carried out in Latin America upon feeding colonized *Lutzomyia longipalpis* sand flies on infected dogs (xenodiagnosis).^5^ ^,^ ^6^ ^,^ ^7^ However, earlier studies in Spain suggested that asymptomatic dogs have the capacity to transmit *L. infantum* to *Phlebotomus perniciosus* sand flies.^32^ Consequently, provided the latter results are the norm, xenodiagnosis studies like those previously used to evaluate the efficacy of antileishmanial drugs still need to be performed after domperidone treatment.^33^ ^,^ ^34^ This will determine whether treated dogs that are subclinically infected could act as reservoir hosts of visceral leishmaniasis.

**TABLE Generic:** names of veterinary drugs with QT interval prolongation

Albuterol	Dopamine	Isoproterenol	Pseudoephedrine
Amantadine	Ephedrine	Methadone	Quinidine
Amiodarone	Epinephrine	Methylphenidate	Sotalol
Chlorpromazine	Erythromycin	Norepinephrine	Terbutaline
Disopyramide	Felbamate	Ondansetron	
Dobutamine	Granisetron	Procainamide	

Modified from Yap and Camm, 2003.^35^

*General conclusions* - Domperidone usage suggests that dogs benefit from its immunomodulatory effects, leading to protection against *L. infantum* infection or improvement of the clinical condition of infected individuals. Evaluation of the risks of side effects related to age, breed, drug interactions, concomitant endocrinopathies and cardiac fitness of dogs should precede domperidone administration. Veterinarians could make an essential contribution to pharmacovigilance, provided they consistently report to local drug agencies all the side effects observed during their clinical practice. The availability of these reports allows regulators to monitor the safety and efficacy of veterinary products once they reach the market. Importantly, domperidone assessment falls within the realm of “one health” since its utilization has impact on the health of both animal and human populations. Future efforts should be placed in defining the capacity of domperidone-treated dogs to participate as reservoir hosts of visceral leishmaniasis.

Provided domperidone demonstrates to reduce significantly the risk of treated dogs to transmit *L. infantum* to vectors, its large-scale implementation could be considered. Since domperidone acts as an immunostimulant with not known direct activity against the parasite, it sub-utilization would not carry the risk of developing drug resistance of *L. infantum*. In Europe, dog-owners are recommended to use the veterinary product, which is conveniently formulated for animal administration (≈15 €/treatment). In Latin America, the principal advantages are the low cost of the generic drug and its oral administration. The generic drug in Brazil costs approximately 0.08 USD per 10 mg tablet; a 10kg-dog would require 15 tablets per treatment (1.20 USD) with a total cost of 3.60 USD for the three recommended treatments/year. An aspect to consider is the need for additional well-powered trials in dog populations that could confirm the published results and meet the approval requirements of health authorities in each endemic country. While domperidone preliminary data look encouraging, its positive impact depends heavily on the government’s commitment and resources to distribute the drug as well as achieving compliance of dog owners of endemic areas. Nevertheless, removal of free-roaming dogs will keep playing a critical role of control campaigns.
